# Intra-ventral tegmental area HIV-1 Tat_1–86_ attenuates nicotine-mediated locomotor sensitization and alters mesocorticolimbic ERK and CREB signaling in rats

**DOI:** 10.3389/fmicb.2015.00540

**Published:** 2015-06-19

**Authors:** Jun Zhu, Narasimha M. Midde, Adrian M. Gomez, Wei-Lun Sun, Steven B. Harrod

**Affiliations:** ^1^Department of Drug Discovery and Biomedical Sciences, South Carolina College of Pharmacy, University of South Carolina, Columbia, SC, USA; ^2^Department of Psychology, University of South Carolina, Columbia, SC, USA

**Keywords:** HIV-1 Tat, nicotine, locomotor activity, behavioral sensitization, CREB, ERK, rat

## Abstract

Cigarette smoking prevalence in the HIV-positive individuals is profoundly higher than that in the HIV-negative individuals. We have demonstrated that HIV-1 transgenic rats exhibit attenuated nicotine-mediated locomotor activity, altered cAMP response element binding protein (CREB) and extracellular regulated kinase (ERK1/2) signaling in the mesocorticolimbic regions. This study investigated the role of HIV-1 transactivator of transcription (Tat) protein in the alterations of nicotine-mediated behavior and the signaling pathway observed in the HIV-1 transgenic rats. Rats received bilateral microinjection of recombinant Tat_1–86_ (25 μg/side) or vehicle directed at ventral tegmental area (VTA) followed by locomotor testing in response to 13 daily intravenous injections of nicotine (0.05 mg/kg, freebase, once/day) or saline. Further, we examined the phosphorylated levels of CREB (pCREB) and ERK1/2 (pERK1/2) in the prefrontal cortex (PFC), nucleus accumbens (NAc) and VTA. Tat diminished baseline activity in saline control rats, and attenuated nicotine-induced behavioral sensitization. Following repeated saline injection, the basal levels of pERK1 in the NAc and VTA and pERK2 in VTA were lower in the vehicle control group, relative to the Tat group. After repeated nicotine injection, pERK1 in NAc and VTA and pERK2 in VTA were increased in the vehicle group, but not in the Tat group. Moreover, repeated nicotine injections decreased pCREB in the PFC and VTA in the Tat group but not in the vehicle group. Thus, these findings indicate that the direct injection of Tat at the VTA may mediate CREB and ERK activity in response to nicotine-induced locomotor activity.

## Introduction

Although the introduction of efficacious antiretroviral therapies reduces the mortality in HIV infected patients, 50% of these patients still suffer from HIV-1-associated neurocognitive disorders (HAND; Sacktor et al., 2001; Sacktor, 2002; Ellis et al., 2007; McArthur et al., 2010; Heaton et al., 2011). Drug abuse in HIV-positive individuals results in greater neurological impairments and precipitates the development of HAND relative to those HIV-positive individuals who do not abuse drugs (Del Valle et al., 2000; Ferris et al., 2008; Hudson et al., 2010; Nath, 2010). According to the Centers for Disease Control and Prevention (CDC) report about cigarette smoking among adults, the rate of cigarette smoking among HIV positive individuals is threefold greater than that in HIV negative population (CDC, 2007). HIV-positive individuals are more likely to become dependent on nicotine and less likely to quit than HIV-negative individuals (Hershberger et al., 2004; Fuster et al., 2009; Nahvi and Cooperman, 2009). Several studies have demonstrated that cigarette smoking is associated with a more rapid progression to AIDS (Nieman et al., 1993; Crothers et al., 2005; Furber et al., 2007; Zhao et al., 2010) and HIV-1-associated dementia (Burns et al., 1996; Manda et al., 2010). Given the high risk for cigarette-associated morbidity and mortality (Crothers et al., 2005), and the great incidence of HAND in HIV positive individuals, there is a critical need to define the molecular mechanisms underlying the enhanced susceptibility to nicotine dependence in HIV smokers.

While the HIV-1 virus enters the brain and produces proviral DNA in the early stage of HIV-1 infection (Nath and Clements, 2011), antiretroviral agents cannot prevent the production of HIV-1 viral proteins, in the brains of HIV-1 infected patients (McArthur et al., 2010; Nath and Clements, 2011). HIV-1 viral proteins are associated with the persistence of HIV-related neuropathology and subsequent neurocognitive deficits (Frankel and Young, 1998; Power et al., 1998; Brack-Werner, 1999; Johnston et al., 2001). The mesocorticolimbic dopamine (DA) pathway is compromised in HIV-positive individuals with drug abuse (Kumar et al., 2009; Norman et al., 2009; Obermann et al., 2009). Long-term viral protein exposure can accelerate damage in the mesocorticolimbic DA system (Nath et al., 1987; Berger and Arendt, 2000; Koutsilieri et al., 2002) and the motivation pathway of the brain (Wise and Bozarth, 1987; Everitt and Robbins, 2005; Berridge, 2007). Among HIV-1 viral proteins, transactivator of transcription (Tat) protein plays a crucial role in the neurotoxicity and cognitive impairment evident in neuroAIDS (Rappaport et al., 1999). Tat activity is sufficient to impair learning and memory performance (Carey et al., 2012). Tat disrupts DA and glutamine transmission by directly interacting with the DA transporter (Zhu et al., 2009; Midde et al., 2013) and NMDA receptor (Li et al., 2008). Since experimental rodents cannot be infected with HIV-1, several approaches are utilized to study the effects of viral proteins on HIV-1 associated neurobiological and behavioral deficits: (1) rodent brain synaptosomes and *in vitro* exposure to Tat (Wallace et al., 2006; Zhu et al., 2009); (2) direct microinjection of Tat into the brain (Harrod et al., 2008; Ferris et al., 2010; Fitting et al., 2010); (3) transgenic mice that express Tat protein (Kim et al., 2003; Duncan et al., 2008); and (4) the HIV-1 transgenic rat model, which carries a *gag-pol*-deleted HIV-1 provirus regulated by the viral promoter expressing seven of the nine HIV-1 viral proteins (Reid et al., 2001). These experimental models mimic different aspects of viral protein-induced neurotoxicity and pathophysiological changes in the brain, although none of these models fully represent the spectrum of HIV-1 viral protein insult in humans. Intra-accumbal or striatal Tat rats show decreased DA transporter activity (Maragos et al., 2002), decreased DA levels (Cass et al., 2003), and attenuated behavioral sensitization to cocaine (Harrod et al., 2008). Tat transgenic mice exhibit increased DA transporter expression (Perry et al., 2010). HIV-1 transgenic rats show enhanced behavioral sensitization to methamphetamine (Liu et al., 2009; Kass et al., 2010) but attenuated nicotine-mediated behavioral sensitization (Midde et al., 2011). These studies suggest that HIV-1 viral proteins alter dopaminergic pathways that in part, mediate behavioral sensitization.

Nicotine is the key component that mediates the addiction to tobacco smoking. DA neurons in the ventral tegmental area (VTA) and their descending projections to the nucleus accumbens (NAc) and prefrontal cortex (PFC) comprise the mesocorticolimbic DA pathway, which mediates the behavioral and biological effects of nicotine (Kita et al., 1992; Panagis et al., 1996; Laviolette and van der Kooy, 2004). Nicotine activates nicotinic acetylcholine receptors (nAChRs) located throughout the mesocorticolimbic DA system in the brain (Kita et al., 1992; Panagis and Spyraki, 1996; Mansvelder et al., 2002; Laviolette and van der Kooy, 2004). The VTA has been demonstrated to be critical for nicotine-induced plasticity (Fu et al., 2000; Mansvelder et al., 2002; Laviolette and van der Kooy, 2004) and play a critical role in nicotine-mediated behavior (Corrigall et al., 1994; Ferrari et al., 2002). The extracellular regulated protein kinase (ERK) and its downstream transcriptional signaling protein, the cyclic AMP response element-binding protein (CREB), appear critical for long-term adaptations in individuals who exhibit drug abuse (Berhow et al., 1996; Carlezon et al., 1998; Nestler, 2001; Girault et al., 2007). Acute nicotine treatment increases CREB phosphorylation in the NAc, striatum and VTA (Walters et al., 2005; Jackson et al., 2009), whereas repeated nicotine treatment in mice decreases CREB phosphorylation in the NAc, and nicotine withdrawal increases CREB phosphorylation in the VTA (Brunzell et al., 2003). Therefore, long-term nicotine exposure leads to neural plasticity in intracellular signaling through the changes of ERK and CREB signaling (Brunzell et al., 2003, 2009; Mineur et al., 2009). Our recent study shows that HIV-1 viral proteins alter basal phosphorylation levels of CREB and ERK and their phosphorylated response to nicotine in the PFC of HIV-1 transgenic rats (Midde et al., 2011). Given the critical role of the VTA in nicotine-mediated plasticity and behavior, along with our recent report that HIV-1 viral proteins altered ERK and CREB activity in mesocorticolimbic areas, the current study examined whether the microinjection of Tat into the VTA altered nicotine-mediated behavioral sensitization and the signaling protein activity. Our results demonstrate that Tat-induced impairment of the VTA would attenuate intravenous nicotine-induced sensitization of locomotor activity in rats.

## Materials and Methods

### Animals

Male Sprague-Dawley Rats (225–250 g) were obtained from Harlan Laboratories, Inc (Indianapolis, IN, USA). All rats were surgically preimplanted with an Intracath IV catheter (22 ga, Becton/Dickinson General Medical Corp., Grand Prairie, TX, USA), which was dorsally implanted port for chronic IV injections (Mactutus et al., 1994). Animals were pair housed in standard polyurethane cages throughout the experiment and provided normal rodent food (ProLab Rat/Mouse/Hamster Chow 3000) and water *ad libitum*. The catheters were flushed daily with 0.2 ml of heparinized (2.5%) saline. The colony was maintained at 21 ± 2°C, 50 ± 10% relative humidity and a 12L:12D cycle with lights on at 0700 h (EST). The animals were maintained according to the National Institute of Health (NIH) guidelines in AAALAC accredited facilities. The experimental protocol was approved by the Institutional Animal Care and Use Committee (IACUC) at the University of South Carolina.

### Drugs

Nicotine hydrogen tartrate salt was purchased from Sigma-Aldrich (St. Louis, MO, USA) and dissolved in sterile saline (0.9% sodium chloride). Nicotine was prepared immediately prior to injection. The nicotine solution was neutralized to pH 7.0 with NaHCO_3_. Nicotine (0.05 mg/kg, freebase) was administered as a bolus IV injection delivered in a volume of 1 ml/kg body weight (15 s), and was followed by flushing (15 s) with 0.2 ml heparinized (2.5%) saline (i.e., the approximate volume of the catheter) once daily for 15 days. The rate of infusion is an important factor that mediates the induction and expression of the drug-induced locomotor sensitization. The proposed injection rate is within the duration shown to produce robust-to-moderate behavioral sensitization (Wallace et al., 1996; Samaha et al., 2002).

### Intra-VTA Tat Microinjection

**FIGURE 1:**
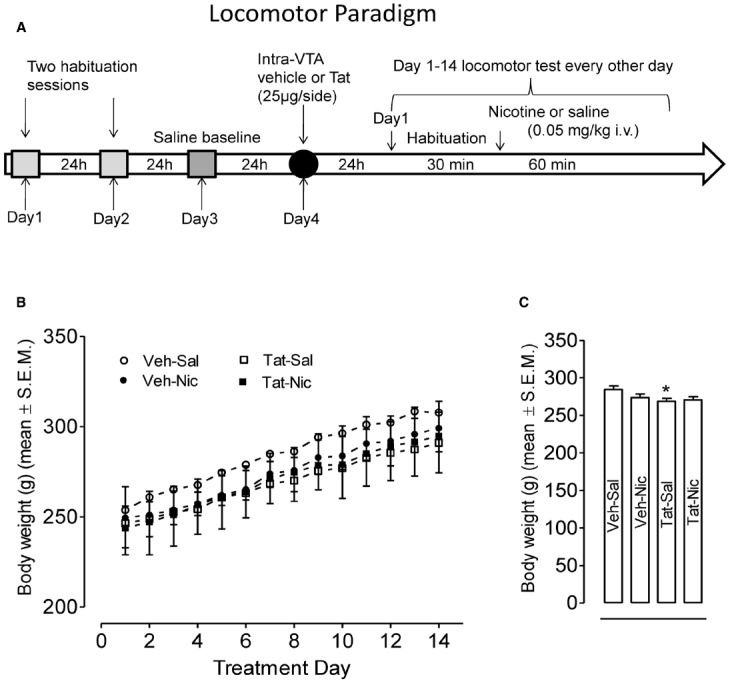
**(A)** Locomotor testing paradigm and time line. **(B)** Body weights of rats with the intra-VTA vehicle and the intra-VTA Tat across daily injection of nicotine (Veh-Nic or Tat-Nic) or saline (Veh-Sal or Tat-Sal). **(C)** Total body weights (mean ± SEM) of rats with the intra-VTA vehicle and the intra-VTA Tat collapsed across 14-day injections of nicotine or saline. *n* = 10–12 rats per group. *p < 0.05 difference in activity between Veh-Sal and Tat-Sal groups.

All rats received bilateral microinjections of Tat or vehicle (0.9% NaCl containing 0.03% L-ascorbic acid) directed at the VTA approximately 24 h after the saline baseline activity session (Figure 1A).

Briefly, rats were anesthetized using a mixture of ketamine hydrochloride and xylazine by intraperitoneal (IP) injection (7.5 mg ketamine/100 g b.wt. and 30 mg xylazine/100 g b.wt.). Rats were then be secured in a stereotaxic apparatus (David Kopf Instruments, Tujunga, CA, USA) where surgery was performed under aseptic conditions. Body temperature was maintained at 37°C by a heating pad coupled to a rectal thermometer (Harvard Apparatus, Holliston, MA, USA). An injection needle (26G) attached to a 1-mL syringe (Hamilton Co., Reno, NV, USA) was filled with either the Tat solution (10 μg/1 μl dissolved in vehicle) or the vehicle (0.9% physiological saline) and positioned in the VTA (A/P, –5.3 mm; M/L, –1.0 mm; D/V, –7.0 mm; bite bar, 0.0 mm). Bilateral injections (25 μg/side) of recombinant Tat_1–86_ were administered. The injection needle remained in position for 5 min before injection of Tat or vehicle, which was infused over a 5-min period using a minipump (Model-310; Stoelting Co., Wood Dale, IL, USA). The injection needle remained in position for an additional 5 min after injection to allow for diffusion into the surrounding tissue.

### Locomotor Activity Procedure

#### Behavioral Apparatus

The activity monitors were square (40 cm × 40 cm) locomotor activity chambers (Hamilton-Kinder Inc., Poway, CA, USA) that detect free movement of animals by infrared photocell interruptions. This equipment uses an infrared photocell grid (32 emitter/detector pairs) to measure locomotor activity. The chambers were converted into round (∼40 cm diameter) compartments by adding clear Plexiglas inserts; photocell emitter/detector pairs were tuned by the manufacturer to handle the extra perspex width. Total horizontal activity represents all beam breaks in the horizontal plane. All activity monitors were located in an isolated room.

#### Habituation

Rats were randomly assigned into four groups: intra-vehicle-saline (Veh-Sal), intra-vehicle-nicotine (Veh-Nic), intra-Tat-saline (Tat-Sal), and intra-Tat-nicotine (Tat-Nic). All rats were habituated to the locomotor activity chambers for two 60-min sessions, once/day prior to intracranial injections. No injections were administered on the habituation days. Twenty four hours after the second habituation session, all rats were habituated to the chambers for 30 min prior to injection, and then injected with saline and placed into the activity chambers for 60-min to measure baseline activity.

#### Pre-Injection Habituation and Nicotine-Induced Behavioral Sensitization

The behavioral sensitization procedure was initiated 24 h after intra-VTA Tat or vehicle injection (Figure 1A). First, all rats received a 30-min habituation period in the testing chamber prior to nicotine (0.35 mg/kg) or saline injection as previously reported (Midde et al., 2011). This was done so that the onset of nicotine’s effects did not overlap with the period that rats showed the most exploratory behavior in the chamber, which was during the first 15 min. Previous research indicates that control rats exhibit asymptotic levels of within-session habituation by 20–30 min, according to similar procedures and use of the same automated chambers (Harrod and Van Horn, 2009; Midde et al., 2011). Rats were administered nicotine or saline (i.v.) every day for a total of 14 days. Locomotor activity was assessed every other day, i.e., on days 1, 3, 5, 7, 9, 11, and 13 for 60 min. On alternate days, nicotine and saline administered in the home cage.

### [^3^H]nicotine Binding Assay

The assay was conducted based on our previously published method (Zhu et al., 1996, 2007). In brief, brain tissues were homogenized with a Polytron in 13 volume ice cold assay buffer containing 50 mM Tris-HCl, 120 mM NaCl, 5 mM KCl, 1 mM MgCl_2_, 2 mM CaCl_2_, pH 7.4. Homogenates were centrifuged at 80,000 g at 4°C for 30 min. Pellets will be resuspended in the same volume of buffer and centrifuged again at 80,000 g at 4°C for 30 min. Final pellets were resuspended in the same volume of buffer and the membrane fraction will be used for binding assays. Saturation binding assays were conducted in duplicate in a final volume of 500 μL, containing membrane homogenate (350–500 μg protein/200 μL). Samples were incubated on ice with 7 concentrations (0.1–5 nM) of [^3^H]nicotine (82 Ci/mmol, Perkin Elmer Life Sciences, Boston, MA, USA) for 90 min. Non-specific binding was determined using 10 μM nicotine.

### Western Blot Analysis

Following the completion of the behavioral study, animals were killed by rapid decapitation 24 h after the last injection with nicotine or saline on day 14, and the brains were removed and dissected in a chilled matrix. The injection needle placement in surgerized animals was visually assessed in brain tissue slices, and subjects with the injection placement outside of the target region were excluded from analysis. PFC, NAc, and VTA were dissected and immediately sonicated on ice in a homogenization buffer containing 20 mM HEPES, 0.5 mM EDTA, 0.1 mM EGTA, 0.4 M NaCI, 5 mM MgCI2, 20% glycerol, 1 mM PMSF, phosphatase inhibitor cocktails I (Sigma, P2850) and protease inhibitors (Sigma, P8340). Samples were centrifuged at 12000 g for 15 min. The supernatant was stored at –80°C. Protein concentrations were determined in duplicate using Bio-Rad DC protein detection reagent. Proteins (30, 10, or 15 μg per sample in the PFC, NAc or VTA) were loaded for ERK, phosphorylated ERK (pERK), CREB and phosphorylated CREB (pCREB) immunoreactivity.

Proteins were separated by 10% SDS-polyacrylamide gel electrophoresis (SDS-PAGE) for 90 min at 150 V, and subsequently transferred to Immobilon-P transfer membranes (Cat # IPVH00010, 0.45 μm pore size; Millipore Co., Bedford, MA, USA) in transfer buffer (50 mM Tris, 250 mM glycine, 3.5 mM SDS) using a Mini Trans-Blot Electrophoretic Transfer Cell (Bio-Rad, Hercules, CA, USA) for 110 min at 72 V. Transfer membranes were incubated with blocking buffer (5% dry milk powder in PBS containing 0.5% Tween 20) for 1 h at room temperature followed by incubation with primary antibodies diluted in blocking buffer overnight at 4°C. Antisera against ERK½ (V114A, Promega, Madison, WI, USA) and pERK½ (SC-16982R, Santa cruz biotechnology, inc, Santa Cruz, CA, USA) were used at a dilution of 1:2000 and 1:1000, respectively. Anti-CREB (9104, Cell signaling, Danvers, MA, USA) and anti-pCREB (9196, Cell signaling, Danvers, MA, USA) antibodies were used at a dilution of 1:1000 and 1:500, respectively. Blots were washed 5 min × 5 times with wash buffer (PBS containing 0.5% Tween 20) at room temperature, and then incubated for 1 h in affinity-purified, peroxidase-labeled, anti-rabbit IgG (1:10000 for ERK½, 1:5000 for pERK½, Jackson ImmunoResearch, West Grove, PA, USA) or 1:2000 anti-mouse IgG (7076, Bio-Rad, Hercules, CA, USA) in blocking buffer for 1 h at room temperature. Blots on the transfer membranes were detected using enhanced chemiluminescence and developed on Hyperfilm (ECL-plus; Amersham Biosciences UK Ltd., Little Chalfont Buckinghamshire UK). After detection and quantification of these proteins, each blot was stripped in 10% of Re-blot plus mild antibody stripping solution (CHEMICON, Temecula, CA, USA) for 20 min at room temperature and reprobed for detection of β-tubulin (sc-9104, Santa cruz biotechnology, inc, Santa Cruz, CA, USA). β-tubulin was used to monitor protein loading among samples. Multiple autoradiographs were obtained using different exposure times, and immunoreactive bands within the linear range of detection were quantified by densitometric scanning using Scion image software (Scion Corp., Frederick, MD, USA).

### Data Analyses

The data are presented as mean values ± standard error of the mean (SEM). The body weights of rats were analyzed with a (2 × 2 × 14) mixed factorial analysis of variance (ANOVA), with protein (vehicle or Tat) and treatment (nicotine or saline) as the between-subject factors, and day as the within-subject factor. Two-factor (protein × treatment) ANOVA was used for analyzing the collapsed body weights. A protein × day × time (2 × 2 × 12) mixed factorial ANOVA was used to analyze data from the two habituation days, and a protein × time (2 × 12) factorial ANOVA was conducted on the saline baseline day. The pre-injection habituation part of the experiment was analyzed using a protein × treatment × day × time (2 × 2 × 7 × 6) ANOVA. The effect of repeated nicotine injection on total horizontal activity was analyzed using a protein × treatment × day × time (2 × 2 × 7 × 12) factorial ANOVA. Protein and treatment were the between-subjects factors, and day and time were the within-subjects factors. To determine the effects of repeated nicotine administration on the activity of signaling proteins (ERK and CREB), separate protein × treatment (2 × 2) factorial ANOVAs were performed on the data from the PFC, NAc, and VTA. Simple effect comparisons were made for *post hoc* analyses. The equilibrium dissociation constant (K_d_) for nicotine binding and the maximal nicotine binding capacity (B_max_) were analyzed by non-linear regression fitting of data using GraphPad Prism 5. Differences for B_max_ and K_d_ values between nicotine-treated and control rats were analyzed using an unpaired *t-*test. All statistical analyses were performed using SPSS (standard version 19.0, Chicago, IL, USA) and differences were considered significant at *p* < 0.05.

## Results

### Effects of Tat or Nicotine on Body Weight

After intra-VTA vehicle or Tat injection, daily body weights across 14-day injections of nicotine or saline were recorded (Figures 1B,C). A protein × treatment × day (2 × 2 × 14) ANOVA revealed significant main effects of protein [*F*(1,39) = 4.2, *p* < 0.05] and day [*F*(13,507) = 271, *p* < 0.001]; however, no other main effects or interaction were significant. Subsequently, a protein × day (2 × 14) ANOVA showed that overall body weights of the intra-VTA vehicle rats were greater than that in the intra-VTA Tat rats within saline controls [*F*(1,19) = 5.02, *p* = 0.037, Figure 1B]. Two-factor (2 × 2) ANOVA analysis revealed a significant main effect of protein [*F*(1,52) = 4.36, *p* = 0.042, Figure 1C]. *Post hoc* analysis showed body weights of the intra-vehicle rats were greater than that in the intra-VTA Tat rats [*F*(1,26) = 6.22, *p* = 0.019, Figure 1C], suggesting that intra-VTA Tat produces a small decrease in rat body weight.

### Habituation and Saline Baseline

**FIGURE 2:**
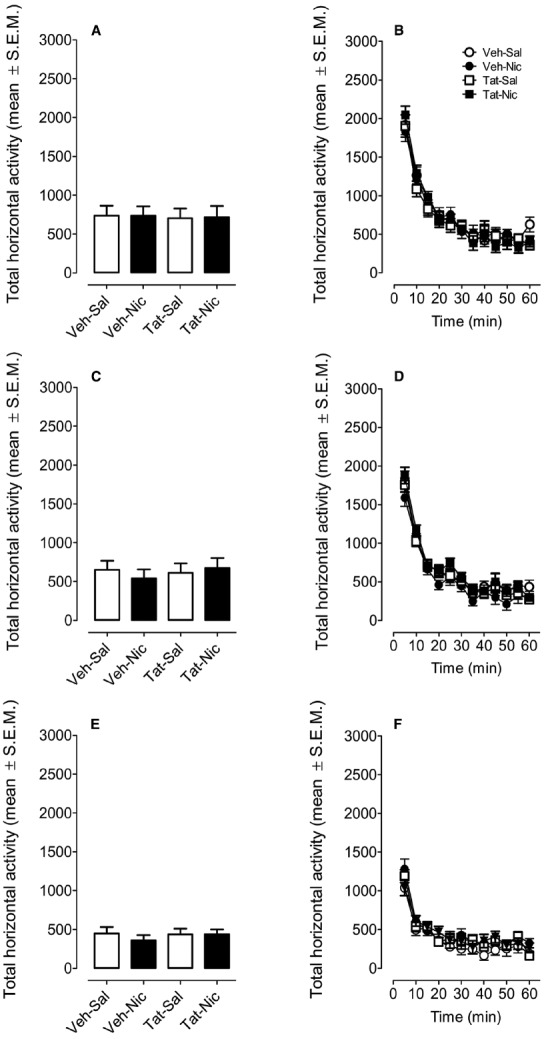
**Locomotor activity during the habituation and the saline baseline sessions.** Rats were randomly assigned to four groups: intra-VTA vehicle and the intra-VTA Tat rats with daily injection of nicotine (Veh-Nic or Tat-Nic) or saline (Veh-Sal or Tat-Sal). **(A,C,E)** Represent the total horizontal activity (mean ± SEM) across the 60-min habituation period. **(B,D,F)** Show the time course of the total horizontal activity (mean ± SEM) during each 5-min interval across the 60-min habituation period. *n* = 10–12 rats per group.

Rats were randomly assigned to four groups: VEH-Sal, VEH-Nic, Tat-Sal and Tat-Nic. Prior to the intracranial Tat microinjection and nicotine injection, all rats were habituated to locomotor activity chambers for 60 min (once/day for 2 days; Figures 2A,C). A protein × day × time ANOVA (2 × 2 × 12) revealed main effects of day [*F*(1,39) = 14.76, *p* < 0.001] and time [*F*(11,429) = 347.79, *p* < 0.001]. Neither main effect of group nor the protein × day × time interaction was significant. All rats exhibited the most activity at first 30-min period of the habituation session and were at asymptote for the remaining 30 min of the session (Figures 2B,D). Figure 2E shows total activity after saline injection. Two-way (protein × time) ANOVA revealed a main effect of time [*F*(11,429) = 61.35, *p* < 0.001]. Neither main effect of protein nor the protein × time interaction was significant (Figure 2F).

### Pre-Injection Habituation

**FIGURE 3:**
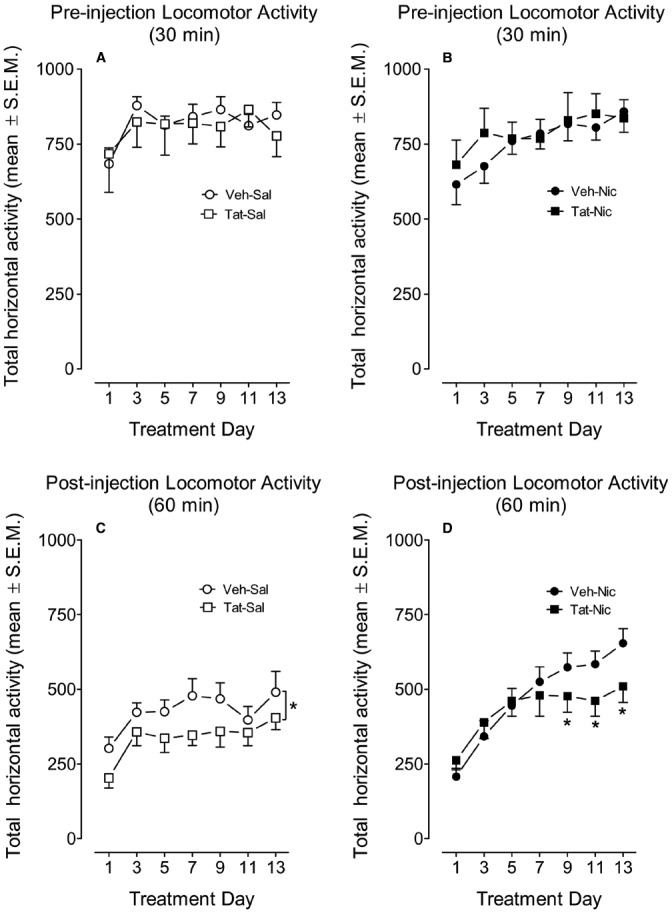
**The time-course data during the behavioral sensitization phase.** Rats with intra-VTA vehicle (Veh) or Tat were intravenously administered nicotine (Nic; 0.35 mg/kg, Veh-Nic, or Tat-Nic) or saline (Veh-Sal or Tat-Sal) on Days 1–13. **(A,B)** Shows the total horizontal activity (mean ± SEM) during the 30-min pre-injection habituation period. **(C,D)** Shows the total horizontal activity (mean ± SEM) during 60 min following nicotine or saline injection. *p < 0.05 difference in activity between the intra-VTA VEH and intra-VTA Tat groups. *n* = 10–12 rats per group.

All rats were placed in the locomotor chambers for 30 min prior to nicotine or saline injection. Figures 3A,B show total horizontal activity during the habituation period across the 13 sessions. A mixed-factor protein × treatment × day × time ANOVA (2 × 2 × 7 × 6) revealed significant main effects of day [*F*(6,234) = 28.86, *p* < 0.001] and time [*F*(5,195) = 477.02, *p* < 0.001], however, no other main effects or interactions were significant.

### Nicotine-Mediated Behavioral Sensitization

The effect of Tat protein on nicotine-mediated locomotor sensitization in rats with and without intra-VTA Tat was determined by measuring total activity following administration of nicotine (0.05 mg/kg, i.v.) or saline (Figures 3C,D). A protein × treatment × day × time ANOVA (2 × 2 × 7 × 6) revealed significant main effects of protein [*F*(1,36) = 3.25, *p* < 0.05], treatment [*F*(1,36) = 4.12, *p* < 0.05], day [*F*(6,216) = 23.9, *p* < 0.001] and time [*F*(6,396) = 332.2, *p* < 0.001]. A significant interactions of treatment × day [*F*(6,216) = 3.68, *p* < 0.01] and treatment × time [*F*(11,396) = 3.18, *p* < 0.01] were found. No significant interactions of protein × treatment and protein × day were detected.

Subsequently, a separate three-factor ANOVA was performed to determine the Tat effects on baseline locomotor activity and nicotine-induced sensitization. In intra-VTA VEH group, significant main effects of day [*F*(6,108) = 16.13, *p* < 0.001] and time [*F*(11,198) = 196.75, *p* < 0.001] were found; however, the main effect of treatment was not significant. The interactions of day × treatment [*F*(6,108) = 4.44, *p* < 0.001] and time × treatment [*F*(6,108) = 4.44, *p* < 0.001] were significant. *Post hoc* analyses showed that nicotine produced hypoactivity on Days 1–3, but enhanced activity on Days 7–13 compared to the respective saline controls (*p* < 0.05). In intra-VTA Tat group, a three-factor ANOVA revealed main effects of treatment [*F*(1,18) = 3.43, *p* < 0.05], day [*F*(6,108) = 8.59, *p* < 0.001] and time [*F*(11,198) = 143, *p* < 0.001]. The interaction of treatment × time [*F*(11,198) = 2.03, *p* < 0.05] was significant; however, the treatment × day interaction was not significant. *Post hoc* analyses showed that no effect of nicotine on activity on Days 1–3, but nicotine produced significant hyperactivity on Days 5–13, relative to the respective saline controls (*p* < 0.05).

Furthermore, separate three-factor ANOVAs were used to determine the differences in locomotor activity between intra-VTA VEH and Tat groups. In the saline control group, the main effects of protein [*F*(1,18) = 3.39, *p* < 0.05], day [*F*(6,108) = 6.34, *p* < 0.001] and time [*F*(11,198) = 166.96, *p* < 0.001] were significant, suggesting that Tat diminishes baseline activity (Figure 3C). No significant interactions of protein × day or protein × time were found. In nicotine-treated groups, the main effect of protein was not significant; however, significant main effects of day [*F*(6,108) = 20.3, *p* < 0.001] and time [*F*(11,198) = 168.6, *p* < 0.001] were found. Moreover, the interaction of protein × day [*F*(6,108) = 2.25, *p* < 0.05] was significant. *Post hoc* tests revealed that nicotine-induced activity was diminished in intra-VTA Tat rats on days 9–13, relative to intra-VTA VEH rats (*p* < 0.05, Figure 3D).

**FIGURE 4:**
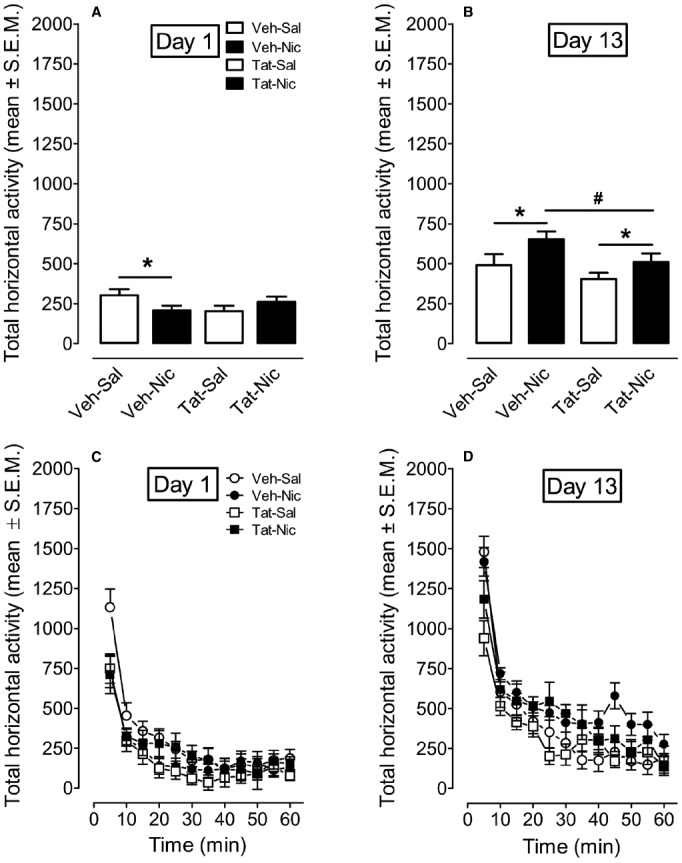
**The time-course data for total horizontal activity during day 1 and day 13 of the behavioral sensitization phase. (A,B)** Show the total horizontal activity (mean ± SEM) across the 60-min session. **(C,D)** Show the time course of the total horizontal activity (mean ± SEM) during each 5-min time interval. *p < 0.05 difference in activity between nicotine-treated and saline control groups. #*p* < 0.05 difference in activity between Veh-Nic and Tat-Nic groups. *n* = 10–12 rats per group.

A protein × treatment × day × time ANOVA was conducted on Day 1 and Day 13 to determine the effect of intra-VTA Tat on nicotine-induced behavioral sensitization (Figure 4). The main effects of protein [*F*(1,36) = 4.04, *p* < 0.05], treatment [*F*(1,36) = 3.0, *p* < 0.05], day [*F*(1,36) = 178.9, *p* < 0.001], and time [*F*(11,396) = 82.02, *p* < 0.001] were significant. Moreover, the interactions of protein × day × treatment [*F*(1,36) = 3.0, *p* < 0.05], protein × time × treatment [*F*(11,396) = 1.84, *p* < 0.05], time × treatment [*F*(11,396) = 3.38, *p* < 0.001] were significant. On day 1, a three-factor ANOVA revealed that a significant interaction of protein × treatment [*F*(1,36) = 5.24, *p* < 0.05]. No significant main effects of protein and treatment were found. Activity was lower in VEH-Nic group than that in VEH-Sal group (*p* < 0.001, Bonferroni *t*-test). Total activity was not different between the Tat-Nic and Tat-Sal groups (*p* > 0.05). Similarly, total activity was not different between VEH-Sal and Tat-Sal groups (*p* > 0.05). On day 13, a three-factor AVONA revealed that significant main effects of protein [*F*(1,36) = 4.46, *p* < 0.05] and treatment [*F*(1,36) = 6.13, *p* < 0.05]. No interaction of protein × treatment was found. *Post hoc* analyses showed that nicotine increased activity in both intra-VTA VEH and Tat groups; however, the nicotine-induced hyperactivity was diminished in intra-VTA Tat group, relative to intra-VTA VEH group (*p* < 0.05; Figure 4B), suggesting that Tat disrupts nicotine-mediated behavioral sensitization.

### Intra-VTA Tat Attenuates Nicotine-Induced Increase of [^3^H]Nicotine Binding in Midbrain

To determine whether intra-VTA Tat affects nAChR density, we measured B_max_ and K_d_ for [^3^H]nicotine binding in the rat midbrain following repeated administration of nicotine or saline (Table 1). Repeated nicotine injections significantly increased B_max_ (21%) in VEH-Nic rats (0.51 ± 0.01), relative to VEH-Sal rats [0.42 ± 0.01, *F*(1,6) = 33.57, *p* < 0.01], suggesting nicotine-induced upregulation of nicotinic receptors. However, the nicotine-induced increase of B_max_ values was not found in intra-VTA Tat rats (0.46 ± 0.01) compared to the respective saline controls (0.50 ± 0.01), indicating that Tat protein attenuates the nicotine-induced augmentation of nicotinic receptors. No difference in K_d_ values was found between groups.

**Table 1:** **Intra tegmental Tat_1–86_ attenuated repeated nicotine-induced upregulation of [^3^H]nicotine binding sites in rat midbrain**.

	**Veh-Sal**	**Veh-Nic**	**Tat-Sal**	**Tat-Nic**
*B*_max_ (fmol/mg protein)	0.42 ± 0.01	0.51 ± 0.01^*^	0.46 ± 0.01	0.50 ± 0.02
*K*_d_ (nM)	1.90 ± 0.37	2.06 ± 0.40	2.16 ± 0.75	1.84 ± 0.52

* Values differing significantly from vehicle control (P < 0.05).

### ERK and CREB Signaling in PFC, NAc, and VTA

**FIGURE 5:**
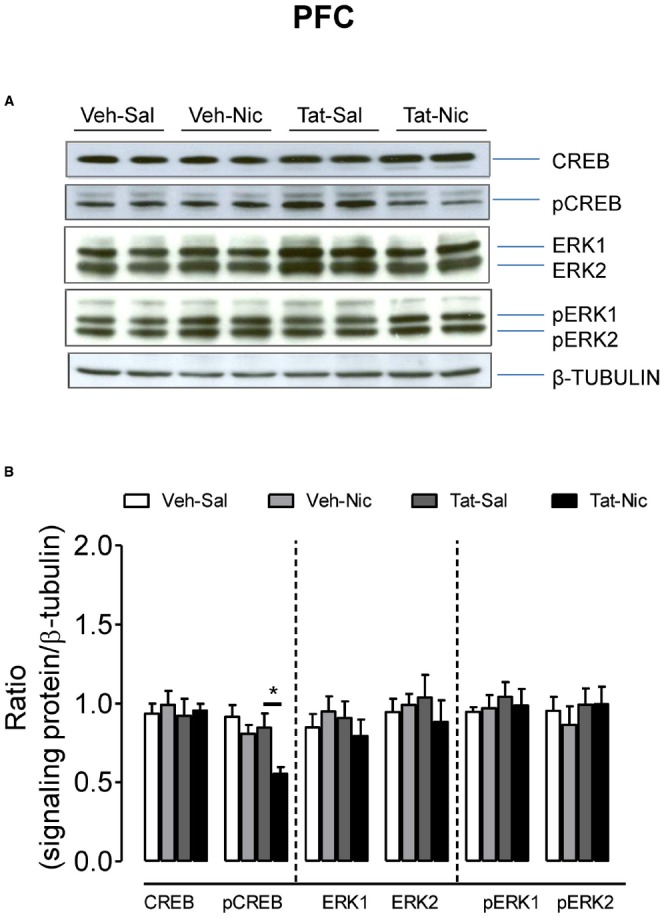
**Levels of ERK and CREB proteins in the PFC of intra-VTA vehicle or Tat rats. (A)** Representative western blots showing the protein density of CREB, pCREB, ERK1/2, pERK1/2 and β-tubulin in the PFC of rats with intra-VTA vehicle (Veh) or Tat following repeated injections of nicotine (Veh-Nic or Tat-Nic) or saline (Veh-Sal or Tat-Sal). **(B)** Total and phosphorylated levels of CREB and ERK1/2. Ratios are presented as the mean percentage of β-tubulin ± SEM *p < 0.05 difference between Tat-Sal and Tat-Nic groups. *n* = 10–12 rats per group.

Separate two-way ANOVAs were performed to determine the effect of Tat on total CREB and ERK1/2, and their phosphorylation levels in the PFC, NAc, and VTA of rats with intra-VTA Tat or vehicle. In PFC, no significant differences in total CREB, ERK1/2, and pERK1/2 were found among the groups (Figure 5). With respect to the ratio of pCREB/β-tubulin, main effects of protein [*F*(1,32) = 13.15, *p* < 0.01] and treatment [*F*(1,32) = 20.21, *p* < 0.01], and interaction of protein × treatment [*F*(1,32) = 4.46, *p* < 0.05] were significant. Repeated administration of nicotine decreased pCREB levels in intra-VTA Tat rats [*F*(1,16) = 19.51, *p* < 0.01], but not in intra-VTA vehicle rats [*F*(1,16) = 3.21, *p* > 0.01] compared to their saline controls.

**FIGURE 6:**
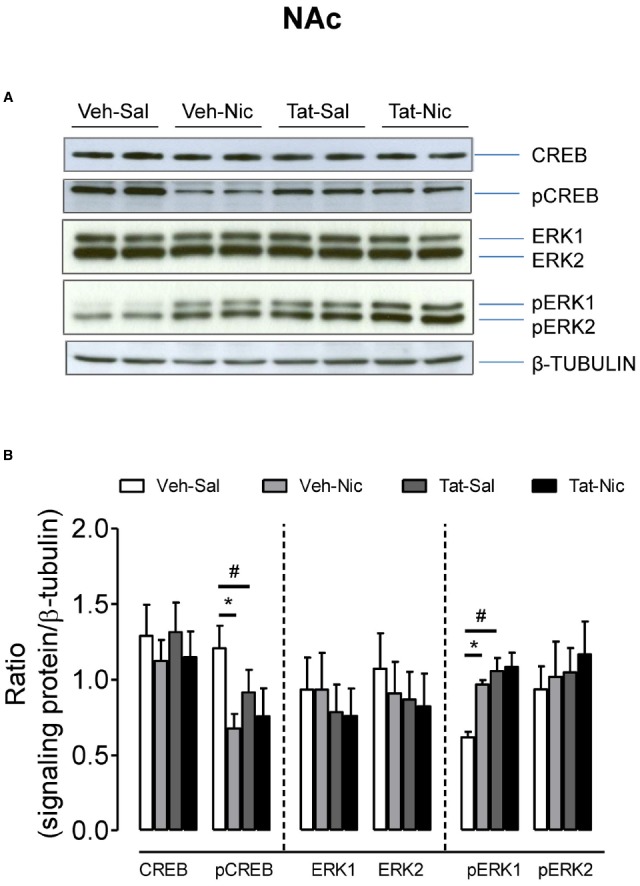
**Levels of ERK and CREB proteins in the NAc of intra-VTA vehicle or Tat rats. (A)** Representative western blots showing the protein density of CREB, pCREB, ERK1/2, pERK1/2, and β-tubulin in the NAc of rats with intra-VTA vehicle (Veh) or Tat following repeated injections of nicotine (Veh-Nic or Tat-Nic) or saline (Veh-Sal or Tat-Sal). **(B)** Total and phosphorylated levels of CREB and ERK1/2. Ratios are presented as the mean percentage of β-tubulin ± SEM *p < 0.05 difference between Veh-Sal and Veh-Nic groups. #*p* < 0.05 difference between Veh-Sal and Tat-Sal groups. *n* = 10–12 rats per group.

In the NAc, as shown in Figure 6, no significant differences were found among the groups in total CREB, ERK1/2, and pREK2. With respect to the ratio of pCREB/β-tubulin, a main effect of treatment [*F*(1,36) = 14.46, *p* < 0.05] and a significant protein × treatment interaction [*F*(1,36) = 4.17, *p* < 0.05] were found. *Post hoc* analysis revealed that pCREB was greater in the Veh-Sal group than that in the Tat-Sal group [*F*(1,18) = 4.29, *p* < 0.05]. Nicotine significantly decreased pCREB in the intra-VTA vehicle group [*F*(1,18) = 14.32, *p* < 0.01] but not in the intra-VTA Tat group relative their saline controls. With respect to the ratio of pERK1/β-tubulin, main effects of protein [*F*(1,20) = 16.64, *p* < 0.05] and treatment [*F*(1,20) = 7.60, *p* < 0.05], and a significant protein × treatment interaction [*F*(1,20) = 5.59, *p* < 0.05] were found (Figure 6). In saline control group, pERK1 was lower in the intra-VTA vehicle group than that in the intra-VTA Tat group [*F*(1,10) = 21.96, *p* < 0.05]. Nicotine significantly increased pERK1 in the intra-VTA vehicle group [*F*(1,10) = 56.10, *p* < 0.01] but not in the intra-VTA Tat group [*F*(1.10) = 0.04, *p* > 0.05] compared with their saline controls.

**FIGURE 7:**
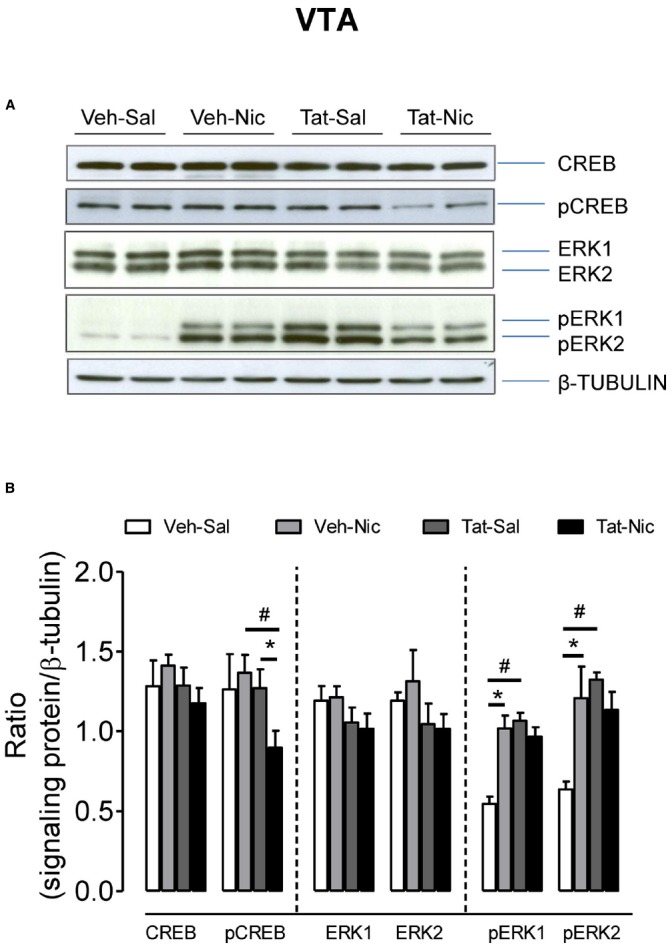
**Levels of ERK and CREB proteins in the VTA of intra-VTA vehicle or Tat rats. (A)** Representative western blots showing the protein density of CREB, pCREB, ERK1/2, pERK1/2, and β-tubulin in the VTA of rats with intra-VTA vehicle (Veh) or Tat following repeated injections of nicotine (Veh-Nic or Tat-Nic) or saline (Veh-Sal or Tat-Sal). **(B)** Total and phosphorylated levels of CREB and ERK1/2. Ratios are presented as the mean percentage of β-tubulin ± SEM *p < 0.05 difference between nicotine-treated and saline control groups. #*p* < 0.05 difference between intra-VTA Veh and intra-VTA Tat groups. *n* = 10–12 rats per group.

Figure 7 shows the ratio of total CREB, pCREB, ERK1/2, and pERK1/2/β-tubulin in the VTA among all groups. Total CREB and ERK1/2 were not different among the groups. For pCREB, two-way ANOVA revealed significant main effects of protein [*F*(1,32) = 5.64, *p* < 0.05] and treatment [*F*(1,32) = 8.67, *p* < 0.01], but the protein × treatment interaction was not significant. Following repeated nicotine administration, pCREB was significantly decreased in the intra-VTA Tat group [*F*(1, 16) = 8.81, *p* < 0.01], but not in the intra-VTA vehicle group [*F*(1, 16) = 1.30, *p* > 0.05] compared to their respective saline controls. The level of pCREB was greater in the intra-VTA vehicle group than that in the intra-VTA Tat group within nicotine treated groups. With respect to pERK1, the main effects of protein [*F*(1,16) = 15.02, *p* < 0.01] and treatment [*F*(1,16) = 9.49, *p* < 0.05] and interaction of protein × treatment [*F*(1,16) = 22.44, *p* < 0.01] were significant. *Post hoc* analysis showed that pERK1 was lower in the intra-VTA vehicle group than that in the intra-VTA Tat group within saline controls [*F*(1,8) = 59.79, *p* < 0.01]. Repeated administration of nicotine increased pERK1 in the intra-VTA vehicle group but not in the intra-VTA Tat group compared to their saline controls [*F*(1,8) = 26.00, *p* < 0.01]. Similarly, regarding to pERK2, the main effect of protein [*F*(1,16) = 6.67, *p* < 0.05] and protein × treatment interaction [*F*(1,16) = 10.28, *p* < 0.05] were significant. *Post hoc* analysis revealed that pERK2 was lower in the intra-VTA vehicle group than that in the intra-VTA Tat group within saline controls [*F*(1,8) = 104.90, *p* < 0.01]. Nicotine significantly increased pERK2 in the intra-VTA vehicle group but not in the intra-VTA Tat group compared to the respective saline controls [*F*(1,8) = 7.84, *p* < 0.05].

## Discussion

The present findings demonstrate that a direct injection of HIV-1 Tat protein into the rat VTA results in neurochemical and behavioral alterations in response to repeated nicotine administration. Specifically, consistent with our recent report as demonstrated in HIV-1 transgenic rats (Midde et al., 2011), intra-VTA Tat diminished locomotor activity under saline control condition and attenuated nicotine-induced behavioral sensitization. Accordingly, intra-VTA Tat decreased the basal levels of pERK1 or pERK2 in the NAc and VTA and attenuated nicotine-mediated changes in pCREB and pERK1/2, suggesting that ERK1/2 and CREB activity may play an important role in Tat-induced changes in locomotion. Together, the findings demonstrate that (1) Tat protein is critical for HIV-1 viral protein-induced neurochemical and behavioral changes in response to nicotine, and (2) the CREB and ERK signaling pathway in the mesocorticolimbic system appears to have played a role in the blunted nicotine-mediated locomotor activity by Tat protein.

The current study demonstrates that rats with intra-VTA Tat exhibited a small decrease in body weight under saline control condition compared to the vehicle control; however, this difference was not observed following repeated nicotine injections. Of interest, our previous work shows that HIV-1 transgenic rats exhibit an inherent reduction of baseline body weight relative to control fisher 344 rats (Midde et al., 2011). The VTA of the midbrain is important for food consumption and body weight (Koyama et al., 2013; Matheny et al., 2014) and plays a critical role in food intake and reward (Mietlicki-Baase et al., 2015). Therefore, one implication of our present findings is that Tat-induced impairment of the DA system within the VTA may, at least in part, result in the reduction of food consumption and body weight noted in HIV-1 transgenic rats (Peng et al., 2010). Although these animals exhibited a lower body weight, they are capable of displaying behavioral sensitization (Liu et al., 2009; Midde et al., 2011), suggesting that the HIV-associated decrease in body weight does not prevent nicotine-induced hyperactivity and the development of behavioral sensitization.

Regarding the behavioral portion of the experiment, the intra-VTA Tat rats exhibited the blunted locomotor response across 60-min behavioral sensitization phases in response to repeated saline injections. Similarly, HIV-1 transgenic rats showed less activity during day 1 of habituations (Midde et al., 2011) and less rearing and head movement activity (Liu et al., 2009), compared to control F344 rats. The blunted locomotor activity appears to be the result of manipulation of DA system by viral proteins (Fink and Smith, 1980), which is evidenced by an increase in D1 receptors (Liu et al., 2009) and a decrease in DAT mRNA (Webb et al., 2010) in the brain of HIV-1 transgenic rats. Opposite effect of D1 receptor expression on baseline locomotor activity has been observed in D1 receptor-deficient mice (El-Ghundi et al., 2010). Thus, these findings further suggest that the diminished locomotor activity is related to neural adaptations produced by Tat protein.

Following repeated nicotine administration, intra-VTA Tat altered the induction of nicotine-mediated behavioral sensitization, as well. First, behavioral sensitization to nicotine was observed in rats with intra-VTA Tat or vehicle, suggesting that intra-VTA Tat animals do not exhibit a deficit in developing behavioral sensitization. Second, compared to saline controls, intra-VTA vehicle rats exhibited lower acute nicotine-mediated activity, whereas acute nicotine had no effect on activity in the intra-VTA Tat rats. No difference in acute nicotine-induced activity between the intra-VTA Tat and intra-VTA vehicle rats was observed. However, following repeated nicotine administration, the intra-VTA Tat rats exhibited the blunted nicotine-mediated locomotor activity during the later days, i.e., 9–13, relative to the intra-VTA vehicle rats. Therefore, intra-VTA Tat rats exhibited less sensitivity to the repeated effects of nicotine, and this deficit may contribute to a manipulation of nAChR-mediated dopamine neurotransmission in the VTA induced by Tat protein. This conclusion is supported by our data showing an attenuation of nicotine-augmented [^3^H]nicotine binding in intra-VTA Tat rats (Table 1). Indeed, bilateral microinjections of cytisine, a nicotinic receptor agonist, into the VTA increase locomotor activity, which is antagonized by either mecamylamine, a nicotinic receptor antagonist, or pimozide, a central dopaminergic antagonist (Museo and Wise, 1990). Furthermore, the nicotine-mediated locomotor activity can be attenuated by ventral tegmental 6-hydroxydopamine lesions (Louis and Clarke, 1998) or by nicotinic receptor antagonists (Clarke and Kumar, 1983). These results suggest that a critical role of nAChR-mediated at the level of the VTA in the nicotine-mediated locomotor activity. Similarly, our previous research found that intracranial injection of Tat into the striatum or NAc of rats increases acute locomotor response to cocaine (Ferris et al., 2010) but attenuated behavioral sensitization to cocaine (Harrod et al., 2008). Together, these findings demonstrate that intra-VTA Tat alters nicotine-mediated behavior, suggesting that cigarette smoking in HIV-positive individuals produces alterations in motivated behavior in response to nicotine due to disrupting the mesocorticolimbic DA system by HIV-1 viral proteins.

The behavioral measure of sensitization reflects the effect of repeated psychostimulant exposure on the mesocorticolimbic DA system. The increase in behavior is hypothesized to correspond to the transition from recreational to compulsive drug intake (Robinson and Berridge, 1993; Pierce and Kalivas, 1997); however, behavioral sensitization is not a measure of drug reward. One potential implication of the attenuated nicotine-mediated sensitized response observed in our previous and current studies is that cigarette smoking by HIV-positive individuals would produce a synergistic influence on motivated behavior due to the interplay of nicotine exposure and viral proteins within the mesocorticolimbic DA system. One caveat is that the current study only tests the nicotine-mediated behavior after Tat exposure, which mimics a synergistic consequence of nicotine and Tat protein in HIV infected tobacco smokers. This study, however, does not address the impact of nicotine-induced neuroadaptations occurring in the brain prior to HIV infection. Nevertheless, the current findings, at least in part, suggest that HIV infection in humans would produce alterations in motivated behavior due to the changes in signaling proteins with the mesocorticolimbic DA system. Given a high tobacco smoking prevalence among HIV-1 positive population, we predict that Tat exposure modulates the abuse liability of nicotine. Determining the role of Tat protein in rodent nicotine self-administration will be an important essential future study.

The second part of the experiment determined ERK and CREB signaling throughout the mesocorticolimbic DA system in rats with intra-VTA Tat or vehicle following repeated nicotine or saline injections. First, in saline control groups, intra-VTA Tat increased basal levels of pERK 1 in the NAc and VTA, and pERK2 in the VTA, compared to vehicle controls, suggesting that the impairment of dopaminergic neuronal activity induced by Tat-mediated neurotoxicity. This conclusion is supported by a previous report showing that methamphetamine-induced neurotoxicity increase in pERK levels (Yan et al., 2014). Second, following repeated nicotine administration, the intra-VTA vehicle group showed increased pERK1 in the NAc and VTA and increased pERK2 in the VTA; however, neither pERK1 nor pERK in the VTA or NAc was altered in the intra-VTA Tat rats, relative to saline controls. Nicotine has been shown to increase the phosphorylation levels of ERK1/2 *in vitro* and *in vivo* (Nakayama et al., 2001; Valjent et al., 2004; Gomez et al., 2015). The attenuation of nicotine-induced pERK1/2 in the intra-VTA Tat group may represent Tat-induced disruption of nicotinic receptor-mediated dopaminergic activity, which is consistent with our previous observation in HIV-1 transgenic rats (Midde et al., 2011). Discrete manipulation of VTA ERK2, using viral-mediated dominant negative mutant of ERK2, diminished cocaine-induced behavioral sensitization (Iniguez et al., 2010). Further, blockage of ERK1/2 activity by SL327, a selective inhibitor of mitogen-activated protein kinase, prevented the induction of locomotion sensitization to either cocaine or amphetamine (Valjent et al., 2005, 2006). In addition, it is possible that the intra-VTA Tat rats have relatively higher basal levels of pERK1/2 under saline control condition, which may produce a ceiling effect on nicotine-mediated pERK1/2, thereby covering the nicotine-induced elevation of pERK1/2 in the intra-VTA Tat group. In accordance, our results show that HIV-1 transgenic rat model exhibited a lower IC_50_ value for [^3^H]nicotine binding with fivefold rightward shift of the nicotine concentration curve in the prefrontal in the midbrain, compared to control Fisher 344 rats (unpublished data) and attenuated chronic nicotine treatment-induced upregulated [^3^H]nicotine binding in the VTA (the current study). Collectively, these findings suggest that long-term Tat exposure can disrupt nicotinic receptor-mediated dopaminergic activity within the mesolimbic area, thereby reducing sensitivity to nicotine. This may, at least in part, explain why HIV-infected patients need to keep smoking and are more likely to become dependent of nicotine.

Previous studies demonstrate that acute Tat exposure *in vitro* produces depolarization on neuronal membrane and increases evoked neuronal firing (Wayman et al., 2015); however, long-term exposure of Tat causes loss of selective populations of neurons *in vitro* and *in vivo* (Cass et al., 2003; Maragos et al., 2003). Furthermore, Tat transgenic mice exhibit marked glial cell activation accompanied by neuronal loss (Kim et al., 2003). These results suggest that Tat may initiate neuronal activity but produces neurotoxic effect on neuronal survivals after a long-term exposure. On the other hand, after chronic administration of nicotine, there was no change in VTA dopamine soma size in mice (Mazei-Robison et al., 2014); however, other study demonstrated that exposure to nicotine *in vitro* increased dendritic arborization and soma size in mesencephalic dopaminergic neurons (Collo et al., 2013). Although there is no report about the synergistic effects of Tat and nicotine on dopaminergic neurons, we hypothesize that intra-VTA Tat can disrupt neuronal activity, such as apoptosis by increasing phosphorylation of ERK activity because the persistent enhancement of ERK activity is associated with cell death (Stanciu et al., 2000; Kulich and Chu, 2001). Given the beneficial outcomes of nicotine on HIV-1 infection-induced neurological deficits (Cao et al., 2013), we speculate that acute nicotine may be beneficial to Tat-induced neuron loss due to Tat-induced transient increase in the neuronal activity; however, long-term exposure of nicotine is unlikely to be beneficial in Tat-induced dysfunction of neuronal activity, such as morphological changes in the VTA neuron. Indeed, a recent study demonstrates that repeated nicotine administration did not alter the learning deficit in HIV-1 transgenic rats (Vigorito et al., 2013). Precise conclusion about the beneficial outcome from nicotine treatment on neurocognitive impairment in HIV-infected patients, however, will require extensive studies of integrity of neurons and synaptic plasticity genes in animals with Tat exposure and Tat plus nicotine treatment.

Repeated nicotine administration decreased pCREB in the NAc but not in PFC and VTA of vehicle controls, whereas the pCREB level was reduced in the PFC and VTA of the intra-VTA Tat group. However, the microinjection of Tat into the VTA did not alter basal pCREB levels. Interestingly, our previous study shows that repeated nicotine injection increased pCREB in F344 control rats but decreased pCREB in HIV-1 transgenic rats, these changes were only observed in the PFC (Midde et al., 2011). The two studies show differential brain regional sensitivity to altered pCREB by viral proteins because two different animal models were used. For example, Tat has higher expression in the PFC compared to other brain regions of HIV-1 transgenic rats (Peng et al., 2010), while direct injection of Tat into the VTA was used in the current study. Nevertheless, our results suggest that nicotine and Tat synergistically affect CREB signaling throughout the mesocorticolimbic system. Tat-induced decrease in CREB activity negatively impact normal function of the mesocorticolimbic DA system (Carlezon et al., 1998), which infers an increase in nicotine award in Tat exposure animals.

## Conclusion

HIV-1 Tat is critical to HIV-1 viral protein-induced alterations of nicotine-mediated behavior and the phosphorylation levels of ERK2 and CREB within the mesocorticolimbic system. The current results suggest that a direct injection of Tat into the VTA enhances basal levels of pERK1/2 in the VTA and NAc, which may result in the low baseline locomotor activity observed in intra-VTA Tat rats. The opposite effects of nicotine on pERK1, pERK2, or pCREB in the VAT and NAc between the intra-VAT Tat and intra-VAT vehicle groups may contribute to the blunted behavioral sensitization to nicotine noted in intra-VAT Tat rats. Determining how Tat influences the nicotine-mediated ERK and CREB signaling in the mesocorticolimbic system will provide insights into understanding molecular mechanisms underlying a high cigarette smoking prevalence among HIV-positive population.

## Author Contributions

Conceived and designed the experiments: JZ and SH. Performed the experiments: JZ, NM, and AG. Analyzed the data: JZ. Technical assistance: SH. Wrote the paper: JZ. Edited the paper: WS, SH.

### Conflict of Interest Statement

The authors declare that the research was conducted in the absence of any commercial or financial relationships that could be construed as a potential conflict of interest.

## References

[B1] Centers for Disease Control and Prevention (CDC). (2007). Cigarette smoking among adults—United States, 2006. MMWR Morb. Mortal. Wkly. Rep. 56, 1157–1161. 17989644

[B2] Berger J. R. Arendt G. (2000). HIV dementia: the role of the basal ganglia and dopaminergic systems. J. Psychopharmacol. 14, 214–221. 10.1177/026988110001400304 11106299

[B3] Berhow M. T. Hiroi N. Nestler E. J. (1996). Regulation of ERK (extracellular signal regulated kinase), part of the neurotrophin signal transduction cascade, in the rat mesolimbic dopamine system by chronic exposure to morphine or cocaine. J. Neurosci. 16, 4707–4715. 8764658 10.1523/JNEUROSCI.16-15-04707.1996 PMC6579030

[B4] Berridge K. C. (2007). The debate over dopamine’s role in reward: the case for incentive salience. Psychopharmacology (Berl.) 191, 391–431. 10.1007/s00213-006-0578-x 17072591

[B5] Brack-Werner R. (1999). Astrocytes: HIV cellular reservoirs and important participants in neuropathogenesis. AIDS 13, 1–22. 10.1097/00002030-199901140-00003 10207540

[B6] Brunzell D. H. Mineur Y. S. Neve R. L. Picciotto M. R. (2009). Nucleus accumbens CREB activity is necessary for nicotine conditioned place preference. Neuropsychopharmacology 34, 1993–2001. 10.1038/npp.2009.11 19212318 PMC2709692

[B7] Brunzell D. H. Russell D. S. Picciotto M. R. (2003). *In vivo* nicotine treatment regulates mesocorticolimbic CREB and ERK signaling in C57Bl/6J mice. J. Neurochem. 84, 1431–1441. 10.1046/j.1471-4159.2003.01640.x 12614343

[B8] Burns D. N. Hillman D. Neaton J. D. Sherer R. Mitchell T. Capps L. (1996). Cigarette smoking, bacterial pneumonia, and other clinical outcomes in HIV-1 infection. Terry Beirn Community Programs for Clinical Research on AIDS. J. Acquir. Immune Defic. Syndr. Hum. Retrovirol. 13, 374–383. 10.1097/00042560-199612010-00012 8948377

[B9] Cao J. Wang S. Wang J. Cui W. Nesil T. Vigorito M. (2013). RNA deep sequencing analysis reveals that nicotine restores impaired gene expression by viral proteins in the brains of HIV-1 transgenic rats. PLoS ONE 8:e68517. 10.1371/journal.pone.0068517 23874651 PMC3712985

[B10] Carey A. N. Sypek E. I. Singh H. D. Kaufman M. J. Mclaughlin J. P. (2012). Expression of HIV-Tat protein is associated with learning and memory deficits in the mouse. Behav. Brain Res. 229, 48–56. 10.1016/j.bbr.2011.12.019 22197678 PMC3580389

[B11] Carlezon W. A. Jr. Thome J. Olson V. G. Lane-Ladd S. B. Brodkin E. S. Hiroi N. (1998). Regulation of cocaine reward by CREB. Science 282, 2272–2275. 10.1126/science.282.5397.2272 9856954

[B12] Cass W. A. Harned M. E. Peters L. E. Nath A. Maragos W. F. (2003). HIV-1 protein Tat potentiation of methamphetamine-induced decreases in evoked overflow of dopamine in the striatum of the rat. Brain Res. 984, 133–142. 10.1016/S0006-8993(03)03122-6 12932847

[B13] Clarke P. B. Kumar R. (1983). Characterization of the locomotor stimulant action of nicotine in tolerant rats. Br. J. Pharmacol. 80, 587–594. 10.1111/j.1476-5381.1983.tb10733.x 6640208 PMC2044999

[B14] Collo G. Bono F. Cavalleri L. Plebani L. Mitola S. Merlo Pich E. (2013). Nicotine-induced structural plasticity in mesencephalic dopaminergic neurons is mediated by dopamine D_3_ receptors and Akt-mTORC1 signaling. Mol. Pharmacol. 83, 1176–1189. 10.1124/mol.113.084863 23543412

[B15] Corrigall W. A. Coen K. M. Adamson K. L. (1994). Self-administered nicotine activates the mesolimbic dopamine system through the ventral tegmental area. Brain Res. 653, 278–284. 10.1016/0006-8993(94)90401-4 7982062

[B16] Crothers K. Griffith T. A. Mcginnis K. A. Rodriguez-Barradas M. C. Leaf D. A. Weissman S. (2005). The impact of cigarette smoking on mortality, quality of life, and comorbid illness among HIV-positive veterans. J. Gen. Intern. Med. 20, 1142–1145. 10.1111/j.1525-1497.2005.0255.x 16423106 PMC1490270

[B17] Del Valle L. Croul S. Morgello S. Amini S. Rappaport J. Khalili K. (2000). Detection of HIV-1 Tat and JCV capsid protein, VP1, in AIDS brain with progressive multifocal leukoencephalopathy. J. Neurovirol. 6, 221–228. 10.3109/13550280009015824 10878711

[B18] Duncan M. J. Bruce-Keller A. J. Conner C. Knapp P. E. Xu R. Nath A. (2008). Effects of chronic expression of the HIV-induced protein, transactivator of transcription, on circadian activity rhythms in mice, with or without morphine. Am. J. Physiol. Regul. Integr. Comp. Physiol. 295, R1680–R1687. 10.1152/ajpregu.90496.2008 18784333 PMC2584859

[B19] El-Ghundi M. B. Fan T. Karasinska J. M. Yeung J. Zhou M. O’Dowd B. F. (2010). Restoration of amphetamine-induced locomotor sensitization in dopamine D1 receptor-deficient mice. Psychopharmacology (Berl.) 207, 599–618. 10.1007/s00213-009-1690-5 19830406 PMC3518283

[B20] Ellis R. Langford D. Masliah E. (2007). HIV and antiretroviral therapy in the brain: neuronal injury and repair. Nat. Rev. Neurosci. 8, 33–44. 10.1038/nrn2040 17180161

[B21] Everitt B. J. Robbins T. W. (2005). Neural systems of reinforcement for drug addiction: from actions to habits to compulsion. Nat. Neurosci. 8, 1481–1489. 10.1038/nn1579 16251991

[B22] Ferrari R. Le Novere N. Picciotto M. R. Changeux J. P. Zoli M. (2002). Acute and long-term changes in the mesolimbic dopamine pathway after systemic or local single nicotine injections. Eur. J. Neurosci. 15, 1810–1818. 10.1046/j.1460-9568.2001.02009.x 12081661

[B23] Ferris M. J. Frederick-Duus D. Fadel J. Mactutus C. F. Booze R. M. (2010). Hyperdopaminergic tone in HIV-1 protein treated rats and cocaine sensitization. J. Neurochem. 115, 885–896. 10.1111/j.1471-4159.2010.06968.x 20796175 PMC4041991

[B24] Ferris M. J. Mactutus C. F. Booze R. M. (2008). Neurotoxic profiles of HIV, psychostimulant drugs of abuse, and their concerted effect on the brain: current status of dopamine system vulnerability in NeuroAIDS. Neurosci. Biobehav. Rev. 32, 883–909. 10.1016/j.neubiorev.2008.01.004 18430470 PMC2527205

[B25] Fink J. S. Smith G. P. (1980). Mesolimbicocortical dopamine terminal fields are necessary for normal locomotor and investigatory exploration in rats. Brain Res. 199, 359–384. 10.1016/0006-8993(80)90695-2 7417789

[B26] Fitting S. Booze R. M. Hasselrot U. Mactutus C. F. (2010). Dose-dependent long-term effects of Tat in the rat hippocampal formation: a design-based stereological study. Hippocampus 20, 469–480. 10.1002/hipo.20648 19489004 PMC3841077

[B27] Frankel A. D. Young J. A. (1998). HIV-1: fifteen proteins and an RNA. Annu. Rev. Biochem. 67, 1–25. 10.1146/annurev.biochem.67.1.1 9759480

[B28] Fu Y. Matta S. G. Gao W. Brower V. G. Sharp B. M. (2000). Systemic nicotine stimulates dopamine release in nucleus accumbens: re-evaluation of the role of *N*-methyl-d-aspartate receptors in the ventral tegmental area. J. Pharmacol. Exp. Ther. 294, 458–465. 10900219

[B29] Furber A. S. Maheswaran R. Newell J. N. Carroll C. (2007). Is smoking tobacco an independent risk factor for HIV infection and progression to AIDS? A systemic review. Sex Transm. Infect. 83, 41–46. 10.1136/sti.2005.019505 16923740 PMC2598585

[B30] Fuster M. Estrada V. Fernandez-Pinilla M. C. Fuentes-Ferrer M. E. Tellez M. J. Vergas J. (2009). Smoking cessation in HIV patients: rate of success and associated factors. HIV Med. 10, 614–619. 10.1111/j.1468-1293.2009.00735.x 19659946

[B31] Girault J. A. Valjent E. Caboche J. Herve D. (2007). ERK2: a logical AND gate critical for drug-induced plasticity? Curr. Opin. Pharmacol. 7, 77–85. 10.1016/j.coph.2006.08.012 17085074

[B32] Gomez A. M. Sun W. L. Midde N. M. Harrod S. B. Zhu J. (2015). Effects of environmental enrichment on ERK1/2 phosphorylation in the rat prefrontal cortex following nicotine-induced sensitization or nicotine self-administration. Eur. J. Neurosci. 41, 109–119. 10.1111/ejn.12758 25328101 PMC4285565

[B33] Harrod S. B. Mactutus C. F. Fitting S. Hasselrot U. Booze R. M. (2008). Intra-accumbal Tat1-72 alters acute and sensitized responses to cocaine. Pharmacol. Biochem. Behav. 90, 723–729. 10.1016/j.pbb.2008.05.020 18582493 PMC2703478

[B34] Harrod S. B. Van Horn M. L. (2009). Sex differences in tolerance to the locomotor depressant effects of lobeline in periadolescent rats. Pharmacol. Biochem. Behav. 94, 296–304. 10.1016/j.pbb.2009.09.009 19766134 PMC2766100

[B35] Heaton R. K. Franklin D. R. Ellis R. J. Mccutchan J. A. Letendre S. L. Leblanc S. (2011). HIV-associated neurocognitive disorders before and during the era of combination antiretroviral therapy: differences in rates, nature, and predictors. J. Neurovirol. 17, 3–16. 10.1007/s13365-010-0006-1 21174240 PMC3032197

[B36] Hershberger S. L. Fisher D. G. Reynolds G. L. Klahn J. A. Wood M. M. (2004). Nicotine dependence and HIV risk behaviors among illicit drug users. Addict. Behav. 29, 623–625. 10.1016/j.addbeh.2003.08.016 15050680

[B37] Hudson L. G. Gale J. M. Padilla R. S. Pickett G. Alexander B. E. Wang J. (2010). Microarray analysis of cutaneous squamous cell carcinomas reveals enhanced expression of epidermal differentiation complex genes. Mol. Carcinog. 49, 619–629. 10.1002/mc.20636 20564339 PMC3626076

[B38] Iniguez S. D. Warren B. L. Neve R. L. Russo S. J. Nestler E. J. Bolanos-Guzman C. A. (2010). Viral-mediated expression of extracellular signal-regulated kinase-2 in the ventral tegmental area modulates behavioral responses to cocaine. Behav. Brain Res. 214, 460–464. 10.1016/j.bbr.2010.05.040 20561901 PMC2914184

[B39] Jackson K. J. Mcintosh J. M. Brunzell D. H. Sanjakdar S. S. Damaj M. I. (2009). The role of *α*6-containing nicotinic acetylcholine receptors in nicotine reward and withdrawal. J. Pharmacol. Exp. Ther. 331, 547–554. 10.1124/jpet.109.155457 19644040 PMC2775251

[B40] Johnston J. B. Zhang K. Silva C. Shalinsky D. R. Conant K. Ni W. (2001). HIV-1 Tat neurotoxicity is prevented by matrix metalloproteinase inhibitors. Ann. Neurol. 49, 230–241. 10.1002/1531-8249(20010201)49:2<230::AID-ANA43>3.0.CO;2-O 11220743

[B41] Kass M. D. Liu X. Vigorito M. Chang L. Chang S. L. (2010). Methamphetamine-induced behavioral and physiological effects in adolescent and adult HIV-1 transgenic rats. J. Neuroimmune Pharmacol. 5, 566–573. 10.1007/s11481-010-9221-z 20532992 PMC4899043

[B42] Kim B. O. Liu Y. Ruan Y. Xu Z. C. Schantz L. He J. J. (2003). Neuropathologies in transgenic mice expressing human immunodeficiency virus type 1 Tat protein under the regulation of the astrocyte-specific glial fibrillary acidic protein promoter and doxycycline. Am. J. Pathol. 162, 1693–1707. 10.1016/S0002-9440(10)64304-0 12707054 PMC1851199

[B43] Kita T. Okamoto M. Nakashima T. (1992). Nicotine-induced sensitization to ambulatory stimulant effect produced by daily administration into the ventral tegmental area and the nucleus accumbens in rats. Life Sci. 50, 583–590. 10.1016/0024-3205(92)90370-5 1736029

[B44] Koutsilieri E. Sopper S. Scheller C. Ter Meulen V. Riederer P. (2002). Involvement of dopamine in the progression of AIDS Dementia Complex. J. Neural. Transm. 109, 399–410. 10.1007/s007020200032 11956960

[B45] Koyama S. Kawaharada M. Terai H. Ohkurano M. Mori M. Kanamaru S. (2013). Obesity decreases excitability of putative ventral tegmental area GABAergic neurons. Physiol. Rep. 1, e00126. 10.1002/phy2.126 24303191 PMC3841055

[B46] Kulich S. M. Chu C. T. (2001). Sustained extracellular signal-regulated kinase activation by 6-hydroxydopamine: implications for Parkinson’s disease. J. Neurochem. 77, 1058–1066. 10.1046/j.1471-4159.2001.00304.x 11359871 PMC1868550

[B47] Kumar A. M. Fernandez J. B. Singer E. J. Commins D. Waldrop-Valverde D. Ownby R. L. (2009). Human immunodeficiency virus type 1 in the central nervous system leads to decreased dopamine in different regions of postmortem human brains. J. Neurovirol. 15, 257–274. 10.1080/13550280902973952 19499455 PMC9618304

[B48] Laviolette S. R. van der Kooy D. (2004). The neurobiology of nicotine addiction: bridging the gap from molecules to behaviour. Nat. Rev. Neurosci. 5, 55–65. 10.1038/nrn1298 14708004

[B49] Li W. Huang Y. Reid R. Steiner J. Malpica-Llanos T. Darden T. A. (2008). NMDA receptor activation by HIV-Tat protein is clade dependent. J. Neurosci. 28, 12190–12198. 10.1523/JNEUROSCI.3019-08.2008 19020013 PMC6671692

[B50] Liu X. Chang L. Vigorito M. Kass M. Li H. Chang S. L. (2009). Methamphetamine-induced behavioral sensitization is enhanced in the HIV-1 transgenic rat. J. Neuroimmune Pharmacol. 4, 309–316. 10.1007/s11481-009-9160-8 19444617

[B51] Louis M. Clarke P. B. (1998). Effect of ventral tegmental 6-hydroxydopamine lesions on the locomotor stimulant action of nicotine in rats. Neuropharmacology 37, 1503–1513. 10.1016/S0028-3908(98)00151-8 9886673

[B52] Mactutus C. F. Herman A. S. Booze R. M. (1994). Chronic intravenous model for studies of drug (Ab)use in the pregnant and/or group-housed rat: an initial study with cocaine. Neurotoxicol. Teratol. 16, 183–191. 10.1016/0892-0362(94)90116-3 8052193

[B53] Manda V. K. Mittapalli R. K. Geldenhuys W. J. Lockman P. R. (2010). Chronic exposure to nicotine and saquinavir decreases endothelial Notch-4 expression and disrupts blood-brain barrier integrity. J. Neurochem. 115, 515–525. 10.1111/j.1471-4159.2010.06948.x 20722969

[B54] Mansvelder H. D. Keath J. R. Mcgehee D. S. (2002). Synaptic mechanisms underlie nicotine-induced excitability of brain reward areas. Neuron 33, 905–919. 10.1016/S0896-6273(02)00625-6 11906697

[B55] Maragos W. F. Tillman P. Jones M. Bruce-Keller A. J. Roth S. Bell J. E. (2003). Neuronal injury in hippocampus with human immunodeficiency virus transactivating protein, Tat. Neuroscience 117, 43–53. 10.1016/S0306-4522(02)00713-3 12605891

[B56] Maragos W. F. Young K. L. Turchan J. T. Guseva M. Pauly J. R. Nath A. (2002). Human immunodeficiency virus-1 Tat protein and methamphetamine interact synergistically to impair striatal dopaminergic function. J. Neurochem. 83, 955–963. 10.1046/j.1471-4159.2002.01212.x 12421368

[B57] Matheny M. Strehler K. Y. King M. Tumer N. Scarpace P. J. (2014). Targeted leptin receptor blockade: role of ventral tegmental area and nucleus of the solitary tract leptin receptors in body weight homeostasis. J. Endocrinol. 222, 27–41. 10.1530/JOE-13-0455 24920667 PMC4066415

[B58] Mazei-Robison M. S. Appasani R. Edwards S. Wee S. Taylor S. R. Picciotto M. R. (2014). Self-administration of ethanol, cocaine, or nicotine does not decrease the soma size of ventral tegmental area dopamine neurons. PLoS ONE 9:e95962. 10.1371/journal.pone.0095962 24755634 PMC3995955

[B59] McArthur J. C. Steiner J. Sacktor N. Nath A. (2010). Human immunodeficiency virus-associated neurocognitive disorders: mind the gap. Ann. Neurol. 67, 699–714. 10.1002/ana.22053 20517932

[B60] Midde N. M. Gomez A. M. Harrod S. B. Zhu J. (2011). Genetically expressed HIV-1 viral proteins attenuate nicotine-induced behavioral sensitization and alter mesocorticolimbic ERK and CREB signaling in rats. Pharmacol. Biochem. Behav. 98, 587–597. 10.1016/j.pbb.2011.03.013 21420997 PMC3091851

[B61] Midde N. M. Huang X. Gomez A. M. Booze R. M. Zhan C. G. Zhu J. (2013). Mutation of tyrosine 470 of human dopamine transporter is critical for HIV-1 Tat-induced inhibition of dopamine transport and transporter conformational transitions. J. Neuroimmune Pharmacol. 8, 975–987. 10.1007/s11481-013-9464-6 23645138 PMC3740080

[B62] Mietlicki-Baase E. G. Reiner D. J. Cone J. J. Olivos D. R. Mcgrath L. E. Zimmer D. J. (2015). Amylin modulates the mesolimbic dopamine system to control energy balance. Neuropsychopharmacology 40, 372–385. 10.1038/npp.2014.180 25035079 PMC4443949

[B63] Mineur Y. S. Brunzell D. H. Grady S. R. Lindstrom J. M. Mcintosh J. M. Marks M. J. (2009). Localized low-level re-expression of high-affinity mesolimbic nicotinic acetylcholine receptors restores nicotine-induced locomotion but not place conditioning. Genes Brain Behav. 8, 257–266. 10.1111/j.1601-183X.2008.00468.x 19077117 PMC2672109

[B64] Museo E. Wise R. A. (1990). Locomotion induced by ventral tegmental microinjections of a nicotinic agonist. Pharmacol. Biochem. Behav. 35, 735–737. 10.1016/0091-3057(90)90316-A 2339162

[B65] Nahvi S. Cooperman N. A. (2009). Review: the need for smoking cessation among HIV-positive smokers. AIDS Educ. Prev. 21, 14–27. 10.1521/aeap.2009.21.3_supp.14 19537951 PMC2704483

[B66] Nakayama H. Numakawa T. Ikeuchi T. Hatanaka H. (2001). Nicotine-induced phosphorylation of extracellular signal-regulated protein kinase and CREB in PC12h cells. J. Neurochem. 79, 489–498. 10.1046/j.1471-4159.2001.00602.x 11701752

[B67] Nath A. (2010). Human immunodeficiency virus-associated neurocognitive disorder: pathophysiology in relation to drug addiction. Ann. N. Y. Acad. Sci. 1187, 122–128. 10.1111/j.1749-6632.2009.05277.x 20201849

[B68] Nath A. Clements J. E. (2011). Eradication of HIV from the brain: reasons for pause. AIDS 25, 577–580. 10.1097/QAD.0b013e3283437d2f 21160414 PMC3681810

[B69] Nath A. Jankovic J. Pettigrew L. C. (1987). Movement disorders and AIDS. Neurology 37, 37–41. 10.1212/WNL.37.1.37 3796836

[B70] Nestler E. J. (2001). Molecular neurobiology of addiction. Am. J. Addict. 10, 201–217. 10.1080/105504901750532094 11579619

[B71] Nieman R. B. Fleming J. Coker R. J. Harris J. R. Mitchell D. M. (1993). The effect of cigarette smoking on the development of AIDS in HIV-1-seropositive individuals. AIDS 7, 705–710. 10.1097/00002030-199305000-00015 8318178

[B72] Norman L. R. Basso M. Kumar A. Malow R. (2009). Neuropsychological consequences of HIV and substance abuse: a literature review and implications for treatment and future research. Curr. Drug Abuse Rev. 2, 143–156. 10.2174/1874473710902020143 19630745 PMC6167747

[B73] Obermann M. Kuper M. Kastrup O. Yaldizli O. Esser S. Thiermann J. (2009). Substantia nigra hyperechogenicity and CSF dopamine depletion in HIV. J. Neurol. 256, 948–953. 10.1007/s00415-009-5052-3 19240951

[B74] Panagis G. Nisell M. Nomikos G. G. Chergui K. Svensson T. H. (1996). Nicotine injections into the ventral tegmental area increase locomotion and Fos-like immunoreactivity in the nucleus accumbens of the rat. Brain Res. 730, 133–142. 10.1016/S0006-8993(96)00432-5 8883897

[B75] Panagis G. Spyraki C. (1996). Neuropharmacological evidence for the role of dopamine in ventral pallidum self-stimulation. Psychopharmacology (Berl.) 123, 280–288. 10.1007/BF02246582 8833421

[B76] Peng J. Vigorito M. Liu X. Zhou D. Wu X. Chang S. L. (2010). The HIV-1 transgenic rat as a model for HIV-1 infected individuals on HAART. J. Neuroimmunol. 218, 94–101. 10.1016/j.jneuroim.2009.09.014 19913921

[B77] Perry S. W. Barbieri J. Tong N. Polesskaya O. Pudasaini S. Stout A. (2010). Human immunodeficiency virus-1 Tat activates calpain proteases via the ryanodine receptor to enhance surface dopamine transporter levels and increase transporter-specific uptake and V_max_. J. Neurosci. 30, 14153–14164. 10.1523/JNEUROSCI.1042-10.2010 20962236 PMC2972730

[B78] Pierce R. C. Kalivas P. W. (1997). A circuitry model of the expression of behavioral sensitization to amphetamine-like psychostimulants. Brain Res. Brain Res. Rev. 25, 192–216. 10.1016/S0165-0173(97)00021-0 9403138

[B79] Power C. Mcarthur J. C. Nath A. Wehrly K. Mayne M. Nishio J. (1998). Neuronal death induced by brain-derived human immunodeficiency virus type 1 envelope genes differs between demented and nondemented AIDS patients. J. Virol. 72, 9045–9053. 9765449 10.1128/jvi.72.11.9045-9053.1998 PMC110321

[B80] Rappaport J. Joseph J. Croul S. Alexander G. Del Valle L. Amini S. (1999). Molecular pathway involved in HIV-1-induced CNS pathology: role of viral regulatory protein, Tat. J. Leukoc. Biol. 65, 458–465. 10204574 10.1002/jlb.65.4.458

[B81] Reid W. Sadowska M. Denaro F. Rao S. Foulke J. Jr. Hayes N. (2001). An HIV-1 transgenic rat that develops HIV-related pathology and immunologic dysfunction. Proc. Natl. Acad. Sci. U.S.A. 98, 9271–9276. 10.1073/pnas.161290298 11481487 PMC55410

[B82] Robinson T. E. Berridge K. C. (1993). The neural basis of drug craving: an incentive-sensitization theory of addiction. Brain Res. Brain Res. Rev. 18, 247–291. 10.1016/0165-0173(93)90013-P 8401595

[B83] Sacktor N. (2002). The epidemiology of human immunodeficiency virus-associated neurological disease in the era of highly active antiretroviral therapy. J. Neurovirol. 8(Suppl. 2), 115–121. 10.1080/13550280290101094 12491162

[B84] Sacktor N. Tarwater P. M. Skolasky R. L. Mcarthur J. C. Selnes O. A. Becker J. (2001). CSF antiretroviral drug penetrance and the treatment of HIV-associated psychomotor slowing. Neurology 57, 542–544. 10.1212/WNL.57.3.542 11502933

[B85] Samaha A. N. Li Y. Robinson T. E. (2002). The rate of intravenous cocaine administration determines susceptibility to sensitization. J. Neurosci. 22, 3244–3250. 11943825 10.1523/JNEUROSCI.22-08-03244.2002 PMC6757529

[B86] Stanciu M. Wang Y. Kentor R. Burke N. Watkins S. Kress G. (2000). Persistent activation of ERK contributes to glutamate-induced oxidative toxicity in a neuronal cell line and primary cortical neuron cultures. J. Biol. Chem. 275, 12200–12206. 10.1074/jbc.275.16.12200 10766856

[B87] Valjent E. Corvol J. C. Trzaskos J. M. Girault J. A. Herve D. (2006). Role of the ERK pathway in psychostimulant-induced locomotor sensitization. BMC Neurosci. 7:20. 10.1186/1471-2202-7-20 16512905 PMC1420315

[B88] Valjent E. Pages C. Herve D. Girault J. A. Caboche J. (2004). Addictive and non-addictive drugs induce distinct and specific patterns of ERK activation in mouse brain. Eur. J. Neurosci. 19, 1826–1836. 10.1111/j.1460-9568.2004.03278.x 15078556

[B89] Valjent E. Pascoli V. Svenningsson P. Paul S. Enslen H. Corvol J. C. (2005). Regulation of a protein phosphatase cascade allows convergent dopamine and glutamate signals to activate ERK in the striatum. Proc. Natl. Acad. Sci. U.S.A. 102, 491–496. 10.1073/pnas.0408305102 15608059 PMC544317

[B90] Vigorito M. Cao J. Li M. D. Chang S. L. (2013). Acquisition and long-term retention of spatial learning in the human immunodeficiency virus-1 transgenic rat: effects of repeated nicotine treatment. J. Neurovirol. 19, 157–165. 10.1007/s13365-013-0154-1 23456952 PMC3643994

[B91] Wallace D. R. Dodson S. Nath A. Booze R. M. (2006). Estrogen attenuates gp120- and tat1-72-induced oxidative stress and prevents loss of dopamine transporter function. Synapse 59, 51–60. 10.1002/syn.20214 16237680

[B92] Wallace D. R. Mactutus C. F. Booze R. M. (1996). Repeated intravenous cocaine administration: locomotor activity and dopamine D2/D3 receptors. Synapse 23, 152–163. 10.1002/(SICI)1098-2396(199607)23:3<152::AID-SYN4>3.0.CO;2-7 8807743

[B93] Walters C. L. Cleck J. N. Kuo Y. C. Blendy J. A. (2005). Mu-opioid receptor and CREB activation are required for nicotine reward. Neuron 46, 933–943. 10.1016/j.neuron.2005.05.005 15953421

[B94] Wayman W. N. Chen L. Persons A. L. Napier T. C. (2015). Cortical consequences of HIV-1 Tat exposure in rats are enhanced by chronic cocaine. Curr. HIV Res. 13, 80–87. 10.2174/0929867322666150311164504 25760043 PMC4896147

[B95] Webb K. M. Aksenov M. Y. Mactutus C. F. Booze R. M. (2010). Evidence for developmental dopaminergic alterations in the human immunodeficiency virus-1 transgenic rat. J. Neurovirol. 16, 168–173. 10.3109/13550281003690177 20337512 PMC3800100

[B96] Wise R. A. Bozarth M. A. (1987). A psychomotor stimulant theory of addiction. Psychol. Rev. 94, 469–492. 10.1037/0033-295X.94.4.469 3317472

[B97] Yan T. Li L. Sun B. Liu F. Yang P. Chen T. (2014). Luteolin inhibits behavioral sensitization by blocking methamphetamine-induced MAPK pathway activation in the caudate putamen in mice. PLoS ONE 9:e98981. 10.1371/journal.pone.0098981 24901319 PMC4047057

[B98] Zhao L. Li F. Zhang Y. Elbourkadi N. Wang Z. Yu C. (2010). Mechanisms and genes involved in enhancement of HIV infectivity by tobacco smoke. Toxicology 278, 242–248. 10.1016/j.tox.2010.09.010 20920546

[B99] Zhu J. Bardo M. T. Green T. A. Wedlund P. J. Dwoskin L. P. (2007). Nicotine increases dopamine clearance in medial prefrontal cortex in rats raised in an enriched environment. J. Neurochem. 103, 2575–2588. 10.1111/j.1471-4159.2007.04951.x 17953677

[B100] Zhu J. Mactutus C. F. Wallace D. R. Booze R. M. (2009). HIV-1 Tat protein-induced rapid and reversible decrease in [^3^H]dopamine uptake: dissociation of [^3^H]dopamine uptake and [^3^H]2β-carbomethoxy-3-β-(4-fluorophenyl)tropane (WIN 35,428) binding in rat striatal synaptosomes. J. Pharmacol. Exp. Ther. 329, 1071–1083. 10.1124/jpet.108.150144 19325033 PMC2683782

[B101] Zhu J. Takita M. Konishi Y. Sudo M. Muramatsu I. (1996). Chronic nicotine treatment delays the developmental increase in brain muscarinic receptors in rat neonate. Brain Res. 732, 257–260. 10.1016/0006-8993(96)00704-4 8891294

